# Supplementary material for: The Effects of Phyllosphere Bacteria on Plant Physiology and Growth of Soybean Infected with Pseudomonas syringae
Source: Plants (Basel). 2022 Oct 7;11(19):2634. doi: 10.3390/plants11192634 (PMC9571934; doi:10.3390/plants11192634)
Supplement: Supplementary file 1 [file plants-11-02634-s001.zip › plants-1902120-supplementary.pdf]

Supplemental Material for Plants special issue “**Beneficial Microorganisms in Sustainable Agriculture**”

The Effects of Phyllosphere-bacteria on Plant Physiology and Growth of Soybean Infected with *Pseudomonas syringae*

Charles Agbavor [1], Babur S. Mirza [1*], D. Alexander Wait [1]

[1] Department of Biology, Missouri State University, Springfield, Missouri, 65897, USA

*Corresponding author: Babur S Mirza
Missouri State University
Department of Biology
901 S. National Ave. Springfield, MO 65897
Phone: 417-836-5062
Fax: 417-836-4204
E-mail: baburmirza@missouristate.edu

Supplementary Figures: S1-S9

Figure S1: A representative image of diseased leaf after 45 days of soybean growth in (A) BC + *Psg* and (B) *Psg* treatment groups.

Figure S2: Quantification of *Psg* cells per leaf disc on day 20 in the BC + *Psg* and *Psg* treatment groups. The number of *Psg* cells per leaf disc was determined for twenty plants per treatment. Three leaves per plant were sampled and six leaf discs per plant were generated. The amount of *Psg* cells per leaf disc was significantly higher in the *Psg* alone treatment compared to the BC + *Psg* treatment on both days (F=1.83; P<0.0001).

Figure S3: Variations in mean soybean plant growth parameters including; dry shoot weight (A), fresh root weight (B), fresh nodule weight (C), and the number of nodules (D) across four different treatment groups on day 20. Treatment had a significant effect on dry shoot (F=28.56; P<0.0001), fresh root weights (F=6.20; P<0.0016), fresh nodule weight (F=8.89; P<0.0002), and nodules number (F=13.36; P<0.0001). The treatments that do not share the same letter are significantly different (alpha = 0.05, Tukey-adjusted). Error bars represent the standard error of the mean.

Figure S4: Variations in mean leaf nitrogen content (%) and chlorophyll concentration ($\mu mol\ m^{-2}$) across four different treatment groups on day 20. Treatment had no effect on the nitrogen content on day 20 (P>0.05). Treatment had a significant effect on chlorophyll concentration on day 20 (F=5.89; P<0.0011). For every plant, the chlorophyll concentration of three leaves was measured and averaged (sample size of 50 plants per treatment). Three distinct areas on every leaf were sampled. The treatments that do not share the same letter are significantly different (alpha = 0.05, Tukey-adjusted). Error bars represent the standard error of the mean.

Figure S5: Variations in mean photosaturated photosynthetic rate A_{max} (A), photosynthetic rate at growth photosynthetically active radiation levels (B), transpiration rate (C) and instantaneous water use efficiency (D) across four different treatment groups on day 20. Treatment had a significant effect on A_{max} (F=10.31; P<0.0001), A_{growth} (F=65.17; P<0.0001), transpiration rate (F=65.62; P<0.0001) and water use efficiency (F=10.30;

P<0.0001) on day 20. The sample size for these variables was 10 plants per treatment. All parameters were measured at ambient PAR levels in the greenhouse as mentioned in the method section. Treatments that do not share the same letter are significantly different (alpha = 0.05, Tukey-adjusted). The error bars represent the standard error of the mean.

Figure S6: Quantification of *Psg* cells per leaf disc on day 45 in the BC + *Psg* and *Psg* treatment groups. The number of *Psg* cells per leaf disc was determined for twenty plants per treatment. Three leaves per plant was sampled and six leaf discs per plant were generated. The amount of *Psg* cells per leaf disc was significantly higher in the *Psg* alone treatment compared to the BC + *Psg* treatment on both days (F=64.25; P<0.0007).

Figure S7: Variations in mean soybean plant growth parameters including; dry shoot weight (A), fresh root weight (B), fresh nodule weight (C), and number of nodules (D) across four different treatment groups on day 45. Treatment had a significant effect on dry shoot (F=40.84; P<0.0001), fresh root weights (F=6.56; P<0.0012), fresh nodule weight (F=13.62; P<0.0001), and nodules number (F=20.24; P<0.0001). The treatments that do not share the same letter are significantly different (alpha = 0.05, Tukey-adjusted). Error bars represent the standard error of the mean.

Figure S8: Variations in mean leaf nitrogen content (%) and chlorophyll concentration (µmol m^{-2}) across four different treatment groups on day 45. Treatment had no effect on the nitrogen content on day 45 (F=8.47; P<0.0002). Treatment had a significant effect on chlorophyll concentration on day 45 (F=12.35; P<0.0001). For every plant, the chlorophyll concentration of three leaves was measured and averaged (sample size of 50 plants per treatment). Three distinct areas on every leaf were sampled. The treatments that do not share the same letter are significantly different (alpha = 0.05, Tukey-adjusted). Error bars represent the standard error of the mean.

Figure S9: Variations in mean photosaturated photosynthetic rate A_{max} (A), photosynthetic rate at growth photosynthetically active radiation levels (B), transpiration rate (C) and instantaneous water use efficiency (D) across four different treatment groups on day 45. Treatment had a significant effect on A_{max} (F=22.83; P<0.0001), A_{growth} (F=89.78; P<0.0001), transpiration rate (F=74.36; P<0.0001) and water use efficiency (F=18.98;

P<0.0001) on day 20. The sample size for these variables were 10 plants per treatment. All parameters were measured at ambient PAR levels in the greenhouse as mentioned in the method section. Treatments that do not share the same letter are significantly different (alpha = 0.05, Tukey-adjusted). The error bars represent the standard error of the mean.

Figure S1

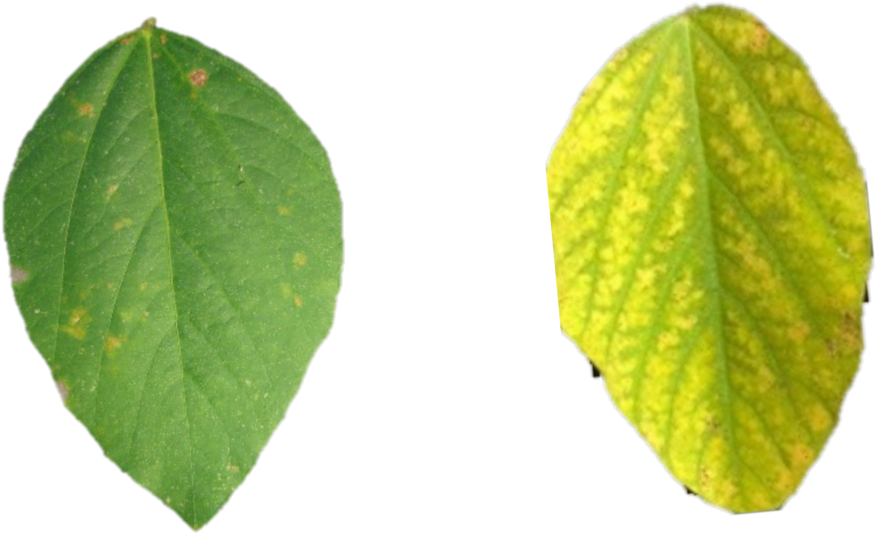

A B

Figure S2

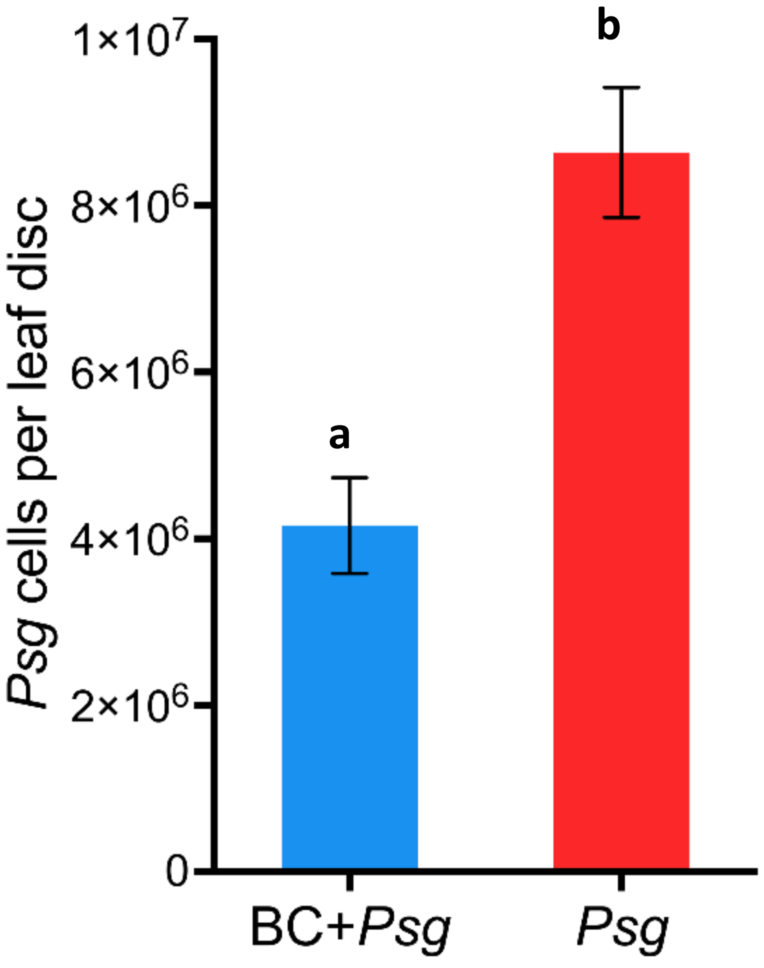

Day 20

Figure S3

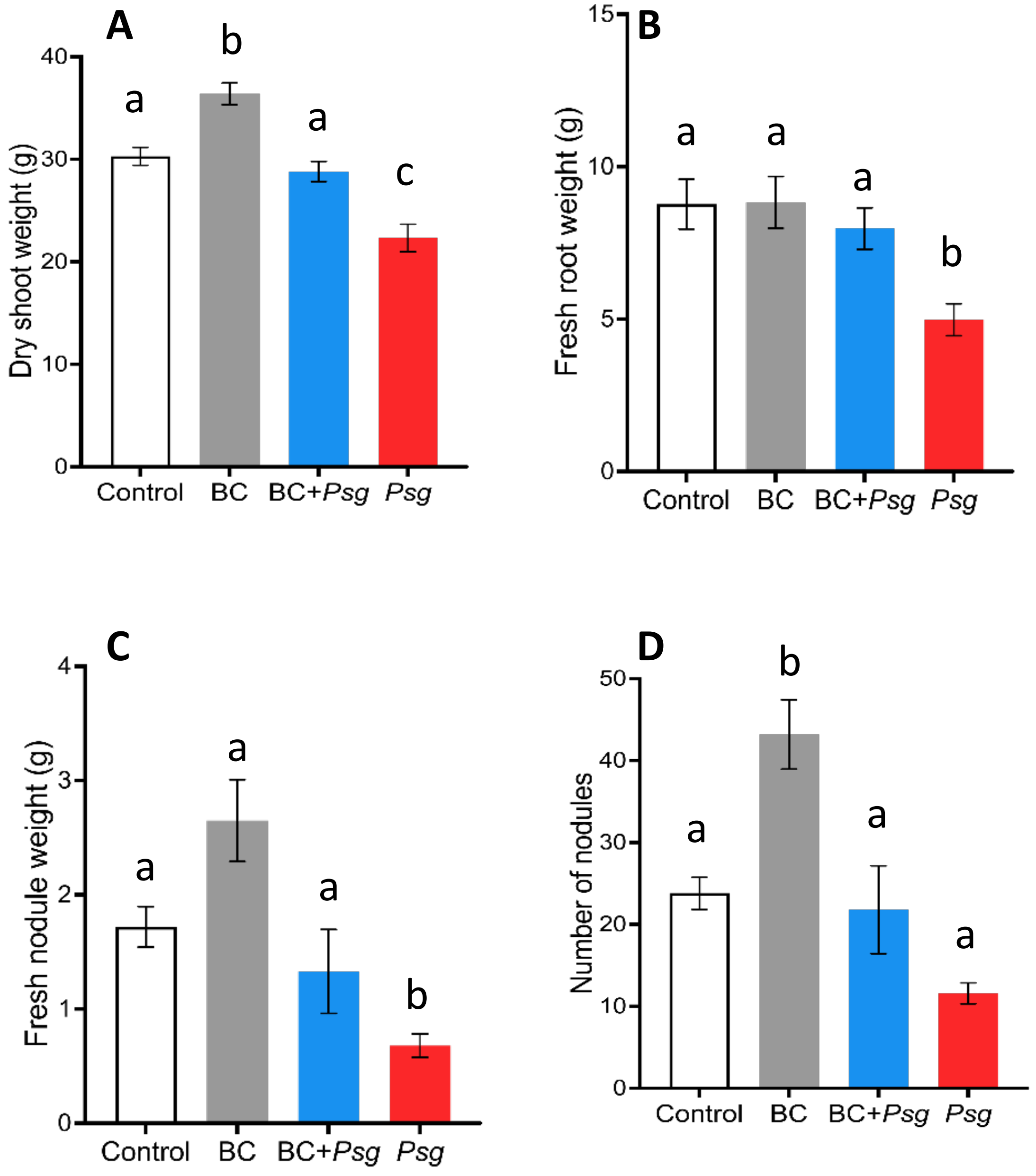

Day 20

Figure S4

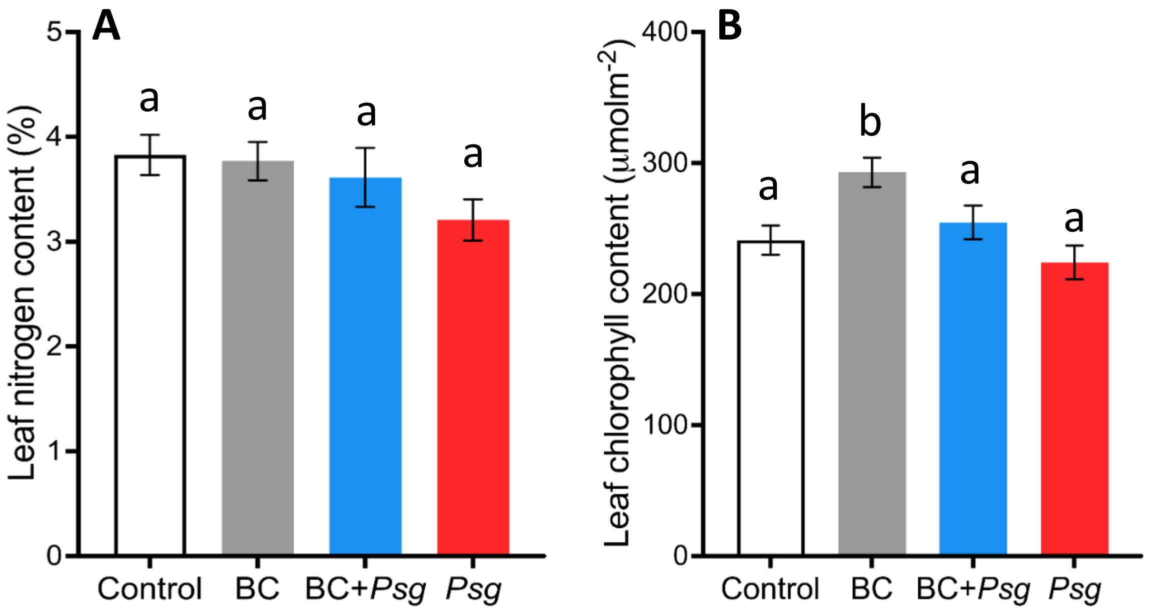

Day 20

Figure S5

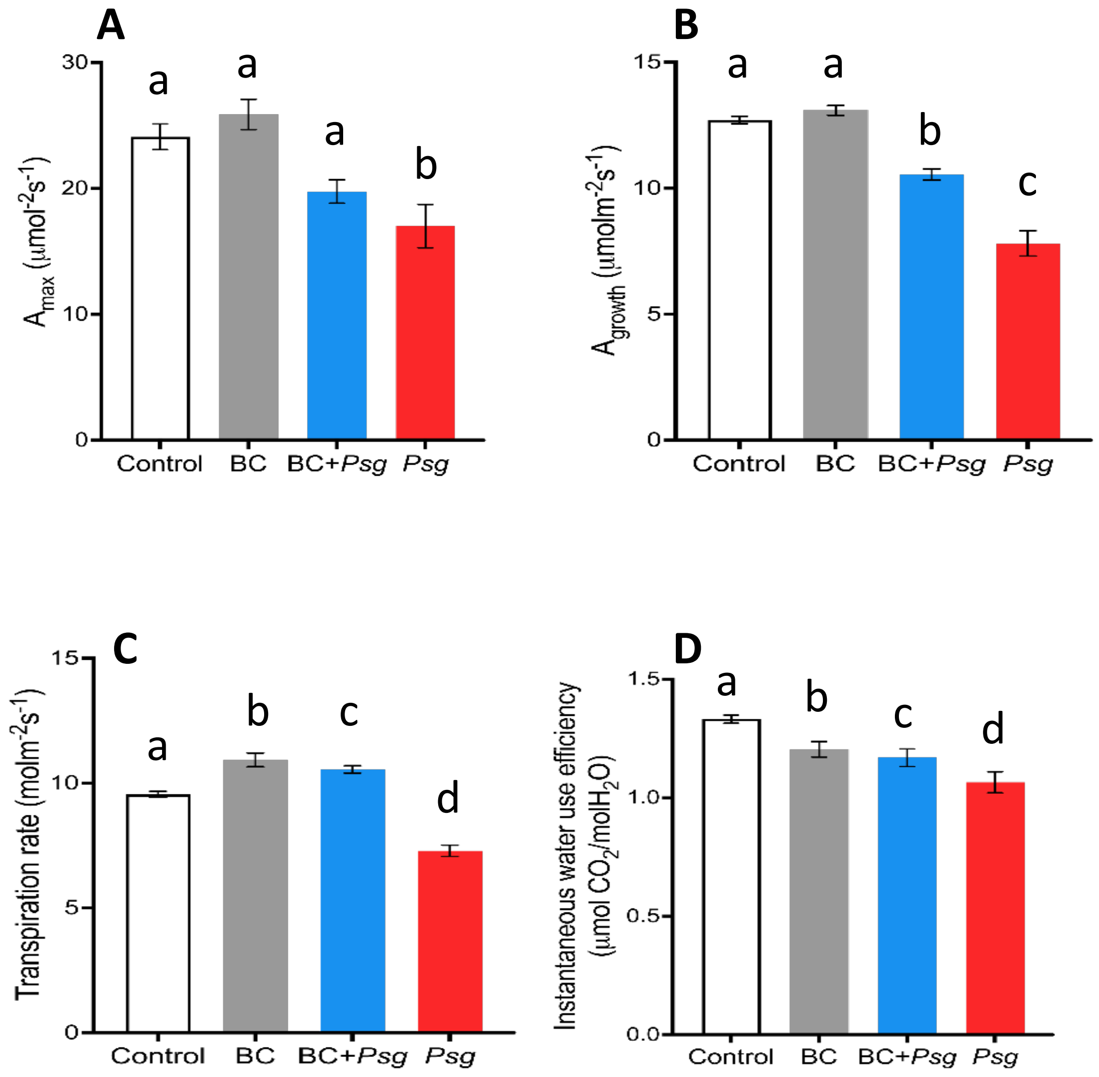

Day 20

Figure S6

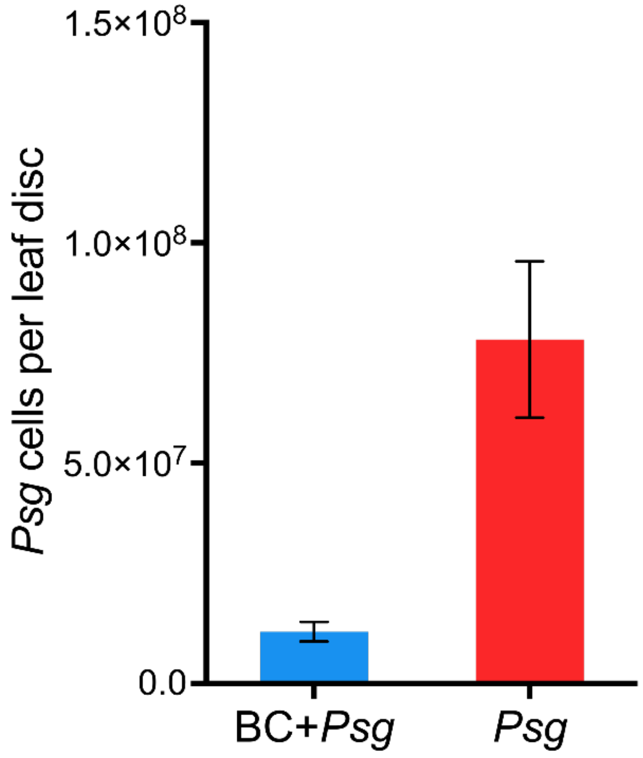

Day 45

Figure S7

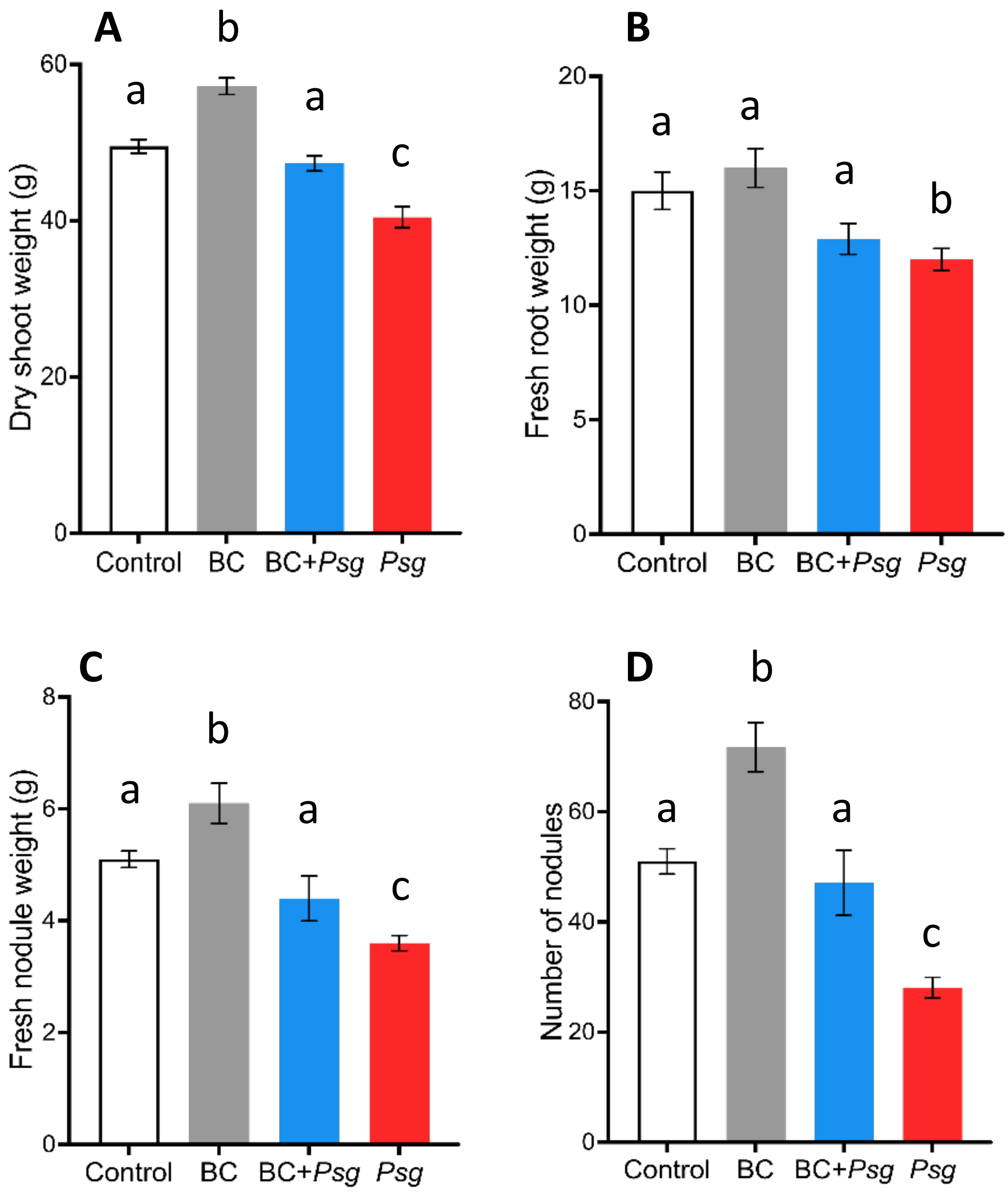

Day 45

Figure S8

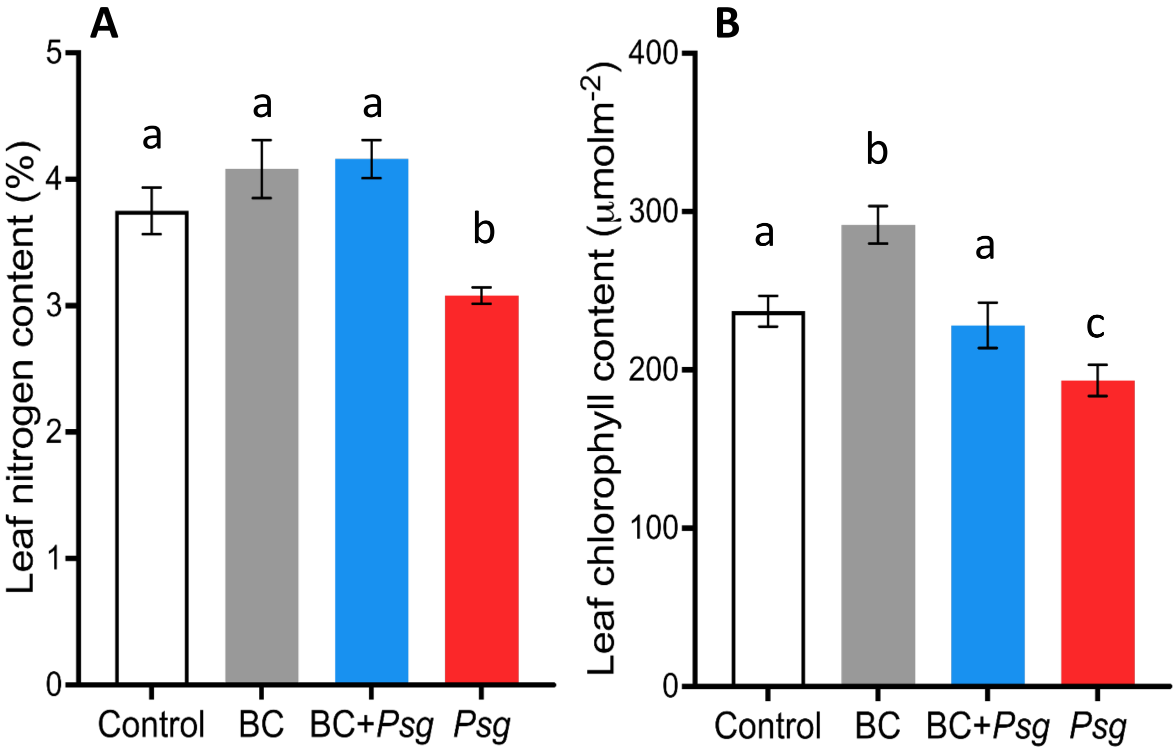

Day 45

Figure S9

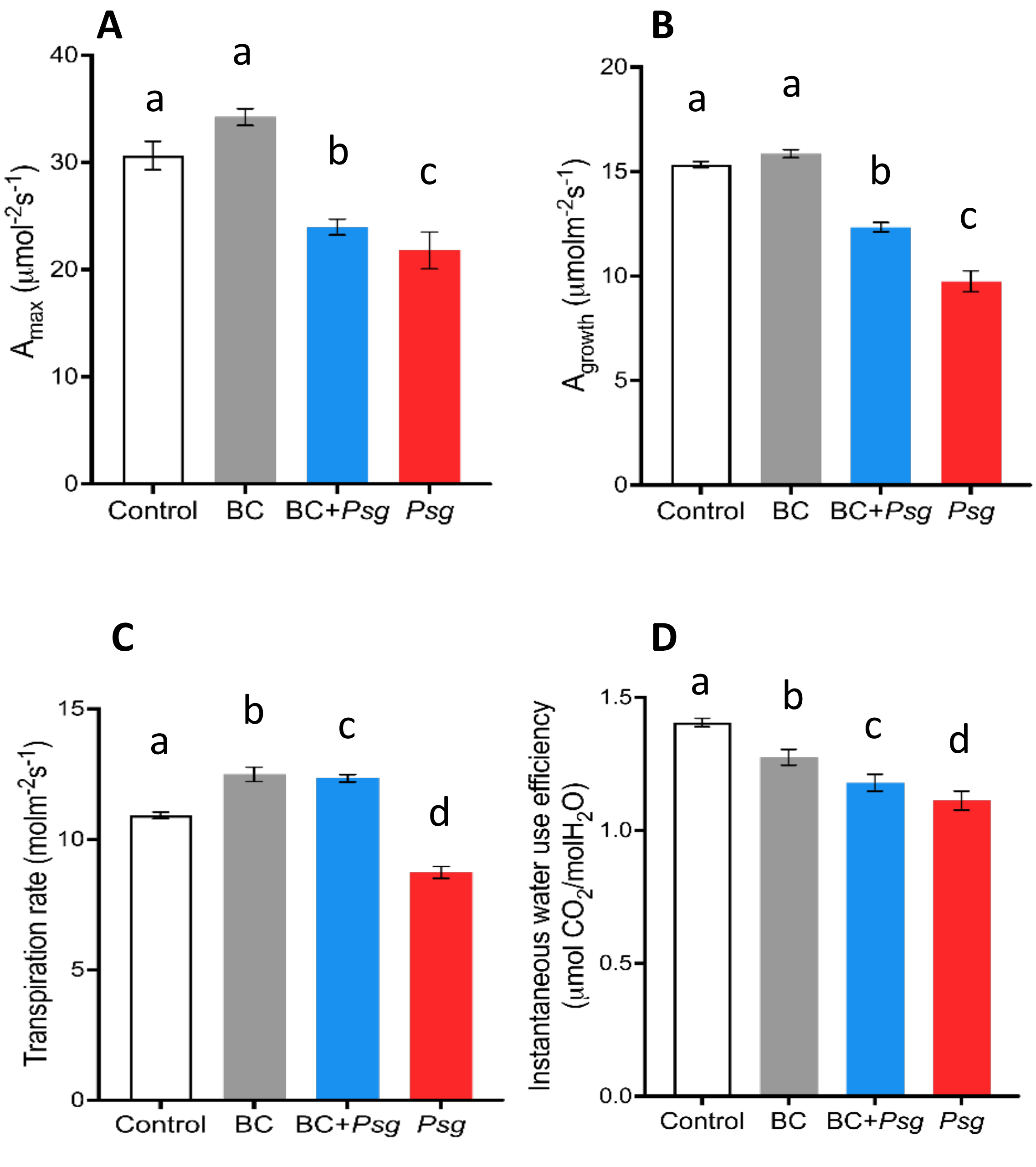

Day 45
